# Postoperative antibiotic therapy after appendectomy in patients with non-perforated appendicitis

**DOI:** 10.22088/cjim.8.2.104

**Published:** 2017

**Authors:** Seyed-Mohammadreza Sadraei-Moosavi, Novin Nikhbakhsh, Ali-asghar Darzi

**Affiliations:** 1 Shahid Beheshti Hospital, Babol University of Medical Sciences, Babol, Iran.; 2 Cancer Research Center, Health Research Institue, Babol University of Medical Sciences, Babol, Iran.; 3 Department of Surgery, Shahid Beheshti Hospital, Babol University of Medical Sciences, Babol, Iran

**Keywords:** Acute appendicitis, Postoperative antibiotics, Prophylactic, Antibiotics, Appendectomy, Surgical site infection, Nonperforated appendicitis

## Abstract

**Background:**

Appendectomy intra-abdominal is the most frequently performed emergency surgery. This study was conducted to determine the role of postoperative antibiotics in reducing surgical site infections (SSIs) and abscess formation after open appendectomy.

**Methods:**

In the Department of Surgery, Shahid Beheshti Hospital, Babol, Iran, from October 2013 to October 2014 one hundred and fifty two patients, who underwent appendectomy for nonperforated appendicitis (NPA) and fulfilled the selection criteria, were randomized into two groups. Group A patients received a single dose of preoperative antibiotics (ceftriaxone and metronidazole) and group B patients received the same regimen, in addition, antibiotics were administered 24 hours postoperatively. Patients of both groups were followed-up for 30 days to assess the postoperative infectious complications.

**Results:**

Both groups comprised 76 patients, as well both groups were compared in baseline characteristics. Statistically, there was no significant difference in rates of SSIs between both groups. None of the patients developed intra-abdominal collection.

**Conclusion:**

Single dose of preoperative antibiotics (ceftriaxone and metronidazole) was sufficient in reducing SSIs after appendectomy for NPA. Postoperative antibiotics did not add an appreciable clinical benefit in these patients.

Appendicitis is the most common cause of acute abdominal pain, requiring surgical intervention and appendectomy is the most frequently performed emergency surgery. Up to 20% of the population has a lifetime risk of developing acute appendicitis (1). Cases of nonperforated appendicitis (NPA) and perforated appendicitis (PA) are categorized as clean contaminated and contaminated, respectively. Several studies have proven the efficacy of preoperative prophylactic antibiotics in reducing postoperative infectious complications after appendectomy (1-5). Therefore, probably all the patients undergoing appendectomy in our hospital are given preoperative prophylactic antibiotics. Patients with perforated appendicitis after appendectomy are universally treated with a variable course of postoperative therapeutic antibiotics because of heavy contamination of wound and peritoneal cavity (6, 7). However, the role of postoperative antibiotics in reducing infectious complications in NPA is still controversial (6-8). The practice of prescribing postoperative antibiotics varies enormously around the globe and no consensus exists on whether postoperative antibiotics are beneficial for preventing infectious complications in NPA. 

Therefore, this trial was conducted to determine the role of postoperative antibiotics in reducing SSIs and intra-abdominal abscess formation after open appendectomy in patients with NPA. 

## Methods

 Between October 2013 and October 2014, a randomized clinical trial was conducted in the Department of Surgery, Shahid Beheshti Hospital, Babol, Iran, after its approval from the Departmental Research Ethics Committee. All patients admitted with acute appendicitis undergoing emergency open appendectomy were considered eligible for this study. Patients who had received antibiotics within 72 hours of admission or who were pregnant or immunocompromised, subjects with diabetes, heart failure, anemia and those patients found to have complicated appendicitis (gangrenous, perforated, appendicular mass or abscess) or normal appendix were excluded. After discharge, the patients who lost to follow-up in the outpatient surgical department were also excluded from the study. Informed consent has been taken from all the patients in this study.

 All the patients received a preoperative dose of ceftriaxone (1g, IV) and metronidazole (500 mg). Open appendectomy was performed by the standard operating technique through right lower quadrant incision (McBurney incision). The wound was closed primarily in all patients after washing with normal saline. After surgery, patients with intraoperative diagnosis of NPA were randomly divided into two groups. Patients who were not given any postoperative antibiotics were included in group A, while group B comprised all patients who received ceftriaxone (1g) and metronidazole (500 mg) up to 24 hours after surgery. Postoperatively all of the patients appendices were sent for histopathological examination.

Patients of both groups were discharged when they were fully mobilized, afebrile, could tolerate normal diet, with evidence of normal bowel activity and had adequate pain control. On discharge, the patients were advised for a follow-up visit in surgical clinic on the 7-14 postoperative days. Futhermore, the patients were advised to report immediately to the emergency department of the hospital in case of fever, tenderness or pus discharge from the wound. Second follow-up visit was arranged a month later after surgery. 

Surgical site infection (SSI) was defined as pus discharge from the wound, redness, tenderness and edema. Intra-abdominal collection was defined as the fluid collection inside the peritoneal cavity confirmed by ultrasound or CT scan. All the infected wounds were managed by laying open the wound, wound toilet with normal saline, and loose packing of the wound and followed by secondary intention. The data regarding demography, clinical symptoms, temperature and CBC on admission, duration of surgery, operative findings, postoperative antibiotics and complications were collected. The date were collected and analyzed by SPSS Version 20. We used the mean and standard deviation for descriptive analysis and to analyze the data we used x2 test and Fisher's exact test, OR or PR with 95%. A p-value of <0.05 was considered as statistically significant. Moreover, this article was registered in IRCT (IRCT2014092219255N1).

## Results

During the study period, 152 patients were admitted with acute appendicitis for open appendectomy. Patients diagnosed to have NPA were randomized divided into two groups. Finally, 152 patients were subjected to statistical analysis. Seventy six patients received only a single dose of preoperative antibiotics (group A); while 76 patients received postoperative antibiotics up to 24 hours after surgery plus preoperative antibiotics (group B). Mean age was 28.45 in group A and 28.30 in group B. There was no statistically significant difference between mean age and hemoglobin in the two groups. One patient in group A and one, patient in group B developed SSIs (table 1). 

**Table 1:** Comparison between group A (no postoperative antibiotics and B (postoperative antibiotics) after surgery)

**Groups** **Variables**	**No postoperative antibiotics (n=76)**	**Postoperative antibiotics (n=76)**	**P-value**
Age (years)	28.45±11.06	28.30±10.70	NS
Hospital Stay	29.65±3.08	30.01±2.98	0.19
Hemoglobin(mg/dl)	13.27±1.49	13.54±1.54	NS
Surgical site infections	1	1	NS
Visits after surgery	2	2	1

NS: non-significant

They were managed by the standard protocol. All the wounds healed in 30 days follow-up. None of the patients developed intra-abdominal collection. There was no significant difference between BMI and the rate of SSIs in two groups. Besides, none of these patients had diabetes and there was no statically significant difference between diabetes and rate of SSIs in two groups in this study. There was no perioperative mortality amongst our patients during this study period.

## Discussion

In the present study, there was no significant difference between the rates of SSIs among the patients with NPA between two groups. Therefore, the addition of postoperative antibiotics with single dose of preoperative antibiotic did not reduce the rate of SSIs in patients with NPA. The incidence of postoperative SSIs after appendectomy in patients with NPA has been reported to range from 0% to 11% (6-11). The stage of the disease process at the time of operation and the use of appropriate prophylactic antibiotics significantly affects the risk for postoperative (7, 10). The efficacy of preoperative antibiotics in reducing the risk of SSI following appendectomy has been well established in the literature (1-5). Only few studies have evaluated the clinical benefits and the disadvantages of administering postoperative antibiotics in addition to adequate preoperative antibiotic prophylaxis (6-11).

In 1995, Liberman et al. reported a high rate of wound infection (11.1%) among the patients who had received only preoperative cefoxitin compared to the patients who were given both pre- and postoperative cefoxitin (1.9%) (9). Nevertheless, they found no infectious complication, in their third group of patients, who had received a single dose of preoperative cefotetan. Thus, they recommended a single dose of preoperative cefotetan as the optimal prophylaxis for NPA. Therefore, the choice of preoperative antibiotic is an important issue, rather than addition of postoperative antibiotics.

Mui et al. conducted a randomized trial on 269 patients to define the optimum duration of prophylactic antibiotics in NPA (6). They found no significant difference in the wound infection rate between three study groups. They concluded that single dose of preoperative antibiotics could adequately prevent the postoperative infectious complications (6). Le et al. compared the patients of NPA who received a single dose of preoperative antibiotics with those who were given postoperative antibiotics in addition to preoperative prophylaxis (7). They observed no significant difference in SSIs rate between the groups (10% vs. 9%). Their wound infection rate was higher than the present study. This difference might be due to type of antibiotics used. Instead of cefoxitin, we used a combination of ceftriaxone and metronidazole. Moreover, high-risk patients (immunocompromised, pregnant) were excluded from this study.

Recently Coakley et al. have compared the outcomes of large number of patients (728 sabjects) treated with antibiotics before and after appendectomy with those who have received only preoperative antibiotics (10). They concluded that the addition of postoperative antibiotics did not reduce infectious complications, also significantly increased morbidity in terms of higher rates of antibiotic-associated diarrhea and clostridium difficile infection. In addition, postoperative antibiotics had significantly prolonged the hospital stay and increased the treatment cost without affording any appreciable clinical benefit (10).

In the present study, there was no significant difference between the rates of SSIs among the patients with NPA between two groups. Hence, the addition of postoperative antibiotics with single dose of preoperative antibiotics did not reduce the rate of SSIs in patients with NPA. These findings are in accordance with other studies (6-11). Both groups were comparable in terms of patient demographics. The high risk patients were excluded from the study. There was no antibiotic-related complication in both groups because of short course of antibiotics. Intra-abdominal abscess formation has rarely been reported after appendectomy in NPA, but it accounts for 2 – 3% of patients in complicated appendicitis (12). This complication was not seen in these patients. In the surgical practice, the supplementary postoperative antibiotics have been used increasingly because of the fear of developing postoperative SSIs. Postoperative antibiotics can not be the substitute of good surgical and aseptic techniques. The overuse of antibiotics is associated with the increased risk of antibiotic- related complications, antibiotic resistant bacteria and cost of care (2, 6-11). For these reasons, the benefits and side effects of antibiotics therapy have to be evaluated carefully. Besides, our results are further strengthened by the recent studies showing that the long-term antibiotic use even in patients with complicated appendicitis does not reduce the postoperative infectious complications (13, 14). There were many limitations in our study. Some of which were: small sample size, no cost assessment between the two groups, no assessment of quality of life and others.

In Conclusion, Single dose of preoperative antibiotics (ceftriaxone and metronidazole) was sufficient in controlling SSIs after appendectomy for NPA. Postoperative antibiotics did not add an appreciable clinical benefit in these patients. As a consequence, surgeons need to update their practice of antibiotic prophylaxis according to the standard guidelines and evidence-based medicine.
